# Chicken (*Gallus gallus*) endogenous retrovirus generates genomic variations in the chicken genome

**DOI:** 10.1186/s13100-016-0085-5

**Published:** 2017-01-24

**Authors:** Jinmin Lee, Seyoung Mun, Dong Hee Kim, Chun-Sung Cho, Dong-Yep Oh, Kyudong Han

**Affiliations:** 1 Department of Nanobiomedical Science & BK21 PLUS NBM Global Research Center for Regenerative Medicine, Dankook University, Cheonan, 330-714 Republic of Korea; 2 Department of Anesthesiology and Pain Management, College of Medicine, Dankook University, Cheonan, 330-714 Republic of Korea; 3 Department of Neurosurgery, College of Medicine, Dankook University, Cheonan, 330-714 Republic of Korea; 4 Gyeongsangbuk-Do Livestock Research Institution, Yeongju, 750-871 Republic of Korea

**Keywords:** Retrotransposon, Full-length GGERV10, Genomic variation, Molecular marker, Incomplete lineage sorting

## Abstract

**Background:**

Transposable elements (TEs) comprise ~10% of the chicken (*Gallus gallus*) genome. The content of TEs is much lower than that of mammalian genomes, where TEs comprise around half of the genome. Endogenous retroviruses are responsible for ~1.3% of the chicken genome. Among them is *Gallus gallus* endogenous retrovirus 10 (GGERV10), one of the youngest endogenous retrovirus families, which emerged in the chicken genome around 3 million years ago.

**Results:**

We identified a total of 593 GGERV10 elements in the chicken reference genome using UCSC genome database and RepeatMasker. While most of the elements were truncated, 49 GGERV10 elements were full-length retaining 5′ and 3′ LTRs. We examined in detail their structural features, chromosomal distribution, genomic environment, and phylogenetic relationships. We compared LTR sequence among five different GGERV10 subfamilies and found sequence variations among the LTRs. Using a traditional PCR assay, we examined a polymorphism rate of the 49 full-length GGERV10 elements in three different chicken populations of the Korean domestic chicken, Leghorn, and *Araucana*. The result found a breed-specific GGERV10B insertion locus in the Korean domestic chicken, which could be used as a Korean domestic chicken-specific marker.

**Conclusions:**

GGERV10 family is the youngest ERV family and thus might have contributed to recent genomic variations in different chicken populations. The result of this study showed that one of GGERV10 elements integrated into the chicken genome after the divergence of Korean domestic chicken from other closely related chicken populations, suggesting that GGERV10 could be served as a molecular marker for chicken breed identification.

**Electronic supplementary material:**

The online version of this article (doi:10.1186/s13100-016-0085-5) contains supplementary material, which is available to authorized users.

## Background

Transposable elements (TEs) are frequently referred to as “junk DNA” in the host genome and compose a major portion of most vertebrate genomes [1]. They are classified as DNA transposons and retrotransposons according to their mobilization methods. DNA transposons integrate into the host genome through a “cut and paste” mechanism but retrotransposons propagate using a “copy and paste” mechanism [2]. TEs have played a role in generating genomic variation, genetic novelty and contributed to speciation and evolutionary transitions in the vertebrate lineage [3]. Several different vertebrate genomes have been sequenced and published [3]. One of them is chicken (*Gallus gallus*) and its size is ~1.2 billion base pairs, which is approximately one third of the size of the most of mammalian including human genome [4, 5]. Unlike most mammalian genomes, TE content is remarkably low in the chicken genome [4–6]. There are various different TE groups in the chicken genome, which include chicken repeat 1 (CR1), long interspersed element 2 (LINE2), endogenous retrovirus (ERV), long terminal repeat (LTR) element, and DNA transposon [4]. Among them, ERVs comprise approximately 1.3% of the chicken genome. This element was originated from exogenous retroviral infection through germ-line cells [4, 7, 8]. ERVs is known to be transmitted vertically in the host genome and propagated through reinfection and retrotransposition events [9]. Avian ERVs are classified into three major exogenous retroviral classes (class I to III), according to *pol* amino acid sequences [10], and consist of four internal coding regions: group-specific antigen (*gag*), protease gene (*pro*), RNA-dependent DNA polymerase gene (*pol*), and envelope gene (*env*), which are flanked by LTRs [11–13]. However, most ERVs are lack of the envelope protein domain due to accumulated mutations (insertion, deletion, and substitution) in the elements and/or negative selection in the host genome [14, 15]. Recently, it was suggested that a retrovirus without *env* gene could be complemented through co-infection with a retrovirus which has a functional *env* [16].

Huda et al. constructed a GGERV phylogenetic tree of fourteen distinct GGERV families based on reverse transcriptase (RT) sequences. GGERV10 element, the youngest ERV family, was integrated into the chicken genome about 0–3 million years ago [8]. Full-length GGERV elements include intact *gag* and *pol* genes, which are necessary for the propagation of the elements. The result of the study showed that GGERV10 family was recently integrated into the chicken genome and proposed that the element could be retrotranspositionally active in the chicken genome.

The LTR sequences of ERV element contain an internal promoter and regulatory sequences (e.g., transcription factor binding site). Therefore, ERVs could alter the expression of host genes by introducing alternative splicing or regulating gene expression in a tissue-specific manner [17]. In fact, it was reported that ERV associated-gene regulation changed the phenotype of its host; *Araucana* lays a blue egg. ERV, locating on the 5’ flanking region of *SLCO1B3* gene in the chicken genome, controls the egg color [18].

In this study, we identified 49 full-length GGERV10 elements in the chicken reference genome (galGal4, Nov. 2011) using a combined method of computational data mining, manual inspection, and experimental validation. Through polymorphism test of the elements, we found that one of them is a Korean breed-specific ERV. This element could be used as a molecular marker for Korean domestic chicken. In sum, we suggest that GGERV10 elements have contributed to the genomic variation of different chicken breeds and could be used as a molecular markers for chicken breed identification.

## Results and discussion

### Identification of GGERV10 insertions

To investigate genomic variation caused by the insertion of GGERV10 family, we computationally extracted 593 putative GGERV10 elements from the chicken (*Gallus gallus*) reference genome, based on RepeatMasker annotation (http://www.repeatmasker.org/cgi-bin/WEBRepeatMasker). Then, we manually inspected them and divided them into three groups: full-length GGERV10 elements, solo-LTRs, and truncated GGERV10 elements. 49, 483, and 61 elements were grouped into full-length GGERV10 elements, solo-LTRs, and truncated GGERV10 elements, respectively. However, the truncated 61 copies were excluded from our data because either or both LTR sequence(s) were missed in them (Table 1). We further examined full-length GGERV10 elements or solo-LTRs, which were probably derived from homologous recombination between LTRs. The remaining 532 GGERV10elements were grouped into five subfamilies, based on their LTR sequence. The LTR sequence variations were annotated by Repbase (http://www.girinst.org/repbase/index.html): GGERV10A, GGERV10B, GGERV10C1, GGERV10C2, and GGERV10D [19]. As shown in Table 2, GGERV10C2 is most abundant while GGERV10B is least abundant in the chicken genome. We examined the chromosomal distribution of GGERV10 and the result showed a high density of the GGERV10 elements on chromosomes 1, 2, and Z. In addition, we calculated the number of GGERV10 insertions per Mbp for each chromosome, and chromosome Z showed the highest insertion/Mbp, shown in Additional file 1: Table S1.

**Table 1:** Summary of GGERV10 elements

Classification	Number of loci
*Computationally extracted GGERV10 loci*	593
Full-length GGERV10 elements	49
Solo-LTR GGERV10 elements	483
Truncated GGERV10 elements	61

**Table 2:** Characterization of GGERV10 subfamilies

Classification		Copy number	Number of full-length	Number of solo-LTRs	Average length of each LTR subfamily
GGERV10 subfamilies	GGERV10A	27	7	20	295
	GGERV10B	25	13	12	382
	GGERV10C1	117	6	111	329
	GGERV10C2	251	10	241	336
	GGERV10D	112	13	99	332

To examine whether the GGERV10 elements have target site preference for their integration, we investigated target site duplications (TSDs) of each of the 532 GGERV10 element including full-length GGERV10 elements and solo-LTRs. TSDs are a hallmark of retrotransposition events. As shown in Additional file 2: Table S2 and Additional file 3: Table S3, there were no target site preferences for GGERV10 insertion.

### Diagnostic sequence characteristics between GGERV10 LTRs

To understand the characteristic of full-length GGERV10 elements, we examined the average length of each LTR sequence. Among the GGERV10 subfamilies, GGERV10B showed the longest LTR sequence with an average of 382 bp. In contrast, the LTR sequence of GGERV10A family was shortest and the averaged size was 295 bp (Table 2). We investigated sequence variations in GGERV10 subfamily by comparing LTR sequences of full-length GGERV10 elements. LTR sequences with a deletion more than 50 bp were excluded for this analysis due to a technical difficulty to align them with other LTR elements. Additional file 4: Figure S1 shows the multiple sequence alignment of LTR sequences (Additional file 5). Interestingly, the full-length GGERV10 elements were divided into two distinct groups, depending on diagnostic sequence characteristics. The first group contained GGERV10A and GGERV10B which shared the ‘E’ region. However, they were distinguished from each other based on ‘A’ and ‘B’ regions. In addition, there was 24-nt duplication (5′-GCGTAGCGAGGGAAACGAGGTGTG-3′) in the GGERV10A subfamily.

GGERV10C1, GGERV10C2, and GGERV10D subfamilies were grouped by sharing the ‘F’ region. We further examined the sequence structure of the second group. The result showed that ‘H’ region was shared between GGERV10C1 and GGERV10C2 subfamilies while the ‘C’ region was shared between GGERV10C1 and GGERV10D subfamilies. However, ‘D’ and ‘G’ regions were unique in GGERV10C2 and GGERV10D subfamilies, respectively. Interestingly, we found a unique sequence feature on GGERV10_76 and GGERV10_205 elements. For example, the 5′ LTR sequence of GGERV10B_76 was matched with the GGERV10D LTR consensus sequence whereas its 3′ LTR sequence was matched with the GGERV10B LTR consensus sequence. The 5′ LTR sequence of GGERV10C2_205 was matched with the GGERV10C2 LTR consensus sequence whereas its 3′ LTR sequence was matched with the GGERV10C1 LTR consensus sequence. Although GGERV10B_76 and GGERV10C2_205 LTR consist of a chimeric structure, we could not find the evidence of a chimeric structure in their body sequence regions (*gag*-*pro*-*pol*-*env*). The GGERV10 elements with a chimeric sequence could be generated by template switching between homologous LTR sequences.

A previous study reported that GGERV10 LTR elements carried fixed dinucleotide terminal inverted repeats, ‘TG’ and ‘CA,’ in the 5′ and 3′ end of their LTR sequences [8]. In this study, we identified GGERV10 LTR-specific terminal inverted repeats, ‘TGTTG’ and ‘CAACA’ at its 5′ and 3′ end, respectively, as shown in Additional file 4: Figure S1.

### Genetic distance between GGERV10 elements

The time of a proviral integration can be estimated based on LTR divergence and intactness of proviral open reading frames (ORFs) [17]. The comparison of LTR sequences is the standard method to estimate the age of full-length ERV insertion [20]. It is well known that the nucleotide difference between the 5′ and 3′ LTR sequences of a single GGERV10 element resulted from point mutations after insertion [21]. Therefore, the nucleotide difference between the 5′ and 3′ LTR sequences could be used to estimate the ERV insertion time [22]. To estimate the age of the GGERV10 subfamilies, we performed the NETWORK analysis [23], based on the evolutionary divergence between all LTR sequences of each subfamily (Additional file 6: Table S4). Using a nucleotide mutation rate of 0.19% per million year (myr) [24], the age of each GGERV10 subfamily was calculated and the result showed that GGERV10B is the youngest GGERV10 subfamily; its estimated age was 3.70 myr.

**Fig. 1:**
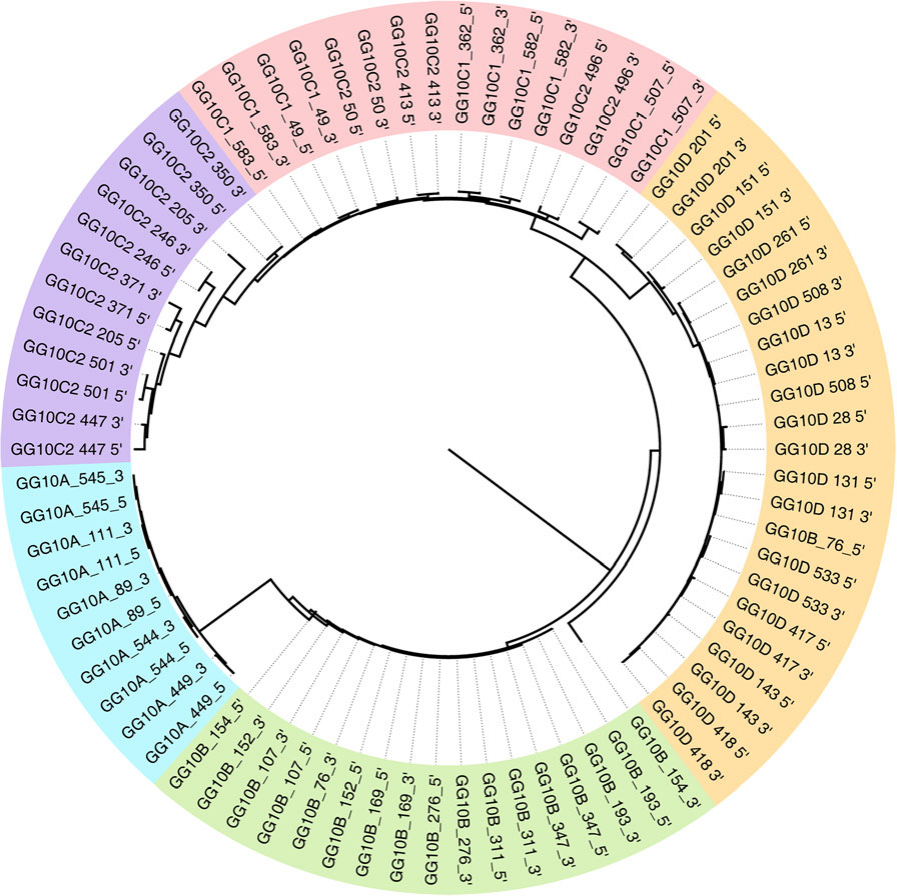
Phylogenetic relationship between the GGERV10 elements. Based on the LTR sequence, neighbor-joining phylogenetic tree of full-length GGERV10 elements was constructed. Evolutionary distances were constructed using the Kimura 2-parameter method [38]. The result of bootstrap calculations (bootstrap value >70%) based on 1,000 replications is shown. The black bar indicates 0.005 nucleotide substitutions per nucleotide position

We also tried to reconstruct the phylogenetic relationships between the full-length GGERV10 LTRs, using a neighbor-joining phylogeny. As we expected, the 5′ and 3′ LTR sequences of each GGERV10 element were highly similar to each other. In addition, our phylogenetic analysis based on 5′ and 3′ LTR sequences of GGERV10 elements grouped them into five different subfamilies, which is consistent with Repbase data [25] (Fig. 1).

### Genomic environment of full-length GGERV10 integration regions

To determine the genomic environment of full-length GGERV10 integration regions, we analyzed the GC content and gene density of genomic regions flanking them (Additional file 2: Table S2). We calculated the GC content in 20-kb windows centered on each GGERV10 locus. The GC content of the flanking regions was, on average, 40.91%, which is lower than the average GC content of the chicken reference genome, 42.92% [26]. It indicates that full-length GGERV10 elements exist in AT-rich regions. We also analyzed the gene density in the 2 Mb of flanking genomic sequences centered on each full-length GGERV10 element. The average gene density of the flanking regions was about 3.83 genes per Mb, which was much lower than that of the chicken genome (an average of 20.41 genes per Mb). The 93.8% (46/49) of full-length GGERV10 elements locate in the intergenic region but only three elements reside in the intronic region. Based on the results, we state that full-length GGERV10 elements preferentially locate in the genomic regions with a high AT content but a low gene density.

### Genomic structure of GGERV10 elements

Structurally or functionally intact ERVs contain *gag*, *pro*/*pol*, and *env* genes but most of the ERVs have not preserved the internal sequences. Over time, integrated ERV copies accumulate nucleotide substitutions or frameshift mutations [27]. In addition, homologous recombination occurs between the two LTRs of each element, leading to a solo-LTR [28].

Using RetroTector10 program [29], we evaluated the genomic structure and function of full-length GGERV10 elements. The program is able to identify open reading frames (ORFs) in chicken ERV elements. The result showed that none of the full-length GGERV10 elements have retained intact *gag*, *pro*/*pol*, and *env* genes. Most of the full-length GGERV10 elements were deficient in *pro*/*pol* and *env* genes. The 31 out of the 49 (63.2%) full-length GGERV10 elements retained the primer-binding site (pbs) and *gag* gene. However, 15 (30.6%) full-length GGERV10 elements contained mutations in the *gag* gene, which were frameshift mutations caused either by insertion or deletion, and the remaining three full-length GGERV10 elements had deficient pbs (Additional file 7: Table S5). Interestingly, all GGERV10B elements contained a polypurine tract in the internal *env* gene, which is served as a primer for the synthesis of the second (plus) DNA strand following reverse transcription [30]. In addition, six out of seven GGERV10A elements had an aspartyl protease (PR) in the internal *pro* gene, which is required for the processing of the Gag precursor, and had a reverse transcriptase in the internal *pol* gene, which is required for reverse transcription of RNA into DNA [31]. Furthermore, we investigated the LTR sequences of full-length GGERV10 elements using TRANSFAC® to identify putative transcription factor binding sites within the LTR sequences. As shown in Additional file 8: Figure S2, the LTR sequences contain 28 different transcription factor binding sites (Additional file 9). The result showed that all of the full-length GGERV10 elements are retrotranspositionally incapable in the chicken genome. However, they might be able to regulate gene expression of the neighboring genes by offering transcription factor binding sites.

### Polymorphism of full-length GGERV10 elements

To check for presence/absence polymorphisms of the 49 full-length GGERV10 elements in the 9 chicken genomic DNA samples (3 for the Korean domestic chicken, 3 for Leghorn, and 3 for *Araucana*), we conducted polymerase chain reaction (PCR) amplification of each full-length GGERV10 locus by using the locus-specific designed primers (Additional file 10: Table S6). The result showed that there are three possible states at a GGERV10 locus: absence of the GGERV10 element, presence of the GGERV10 element, and presence of the solo-LTR generated by the homologous recombination between 5′ and 3′ LTRs. 18.4% of full-length GGERV10 elements were polymorphic in the three different chicken breeds of the Korean domestic chicken, Leghorn, and *Araucana*. The polymorphism level was 28.6% (2/7), 46.1% (6/13), and 7.7% (1/13) for GGERV10A, GGERV10B, and GGERV10D, respectively. In contrast, GGERV10C1 and C2 subfamilies showed no polymorphism in the chicken breeds.

### Molecular markers for identification of chicken breeds

**Fig. 2:**
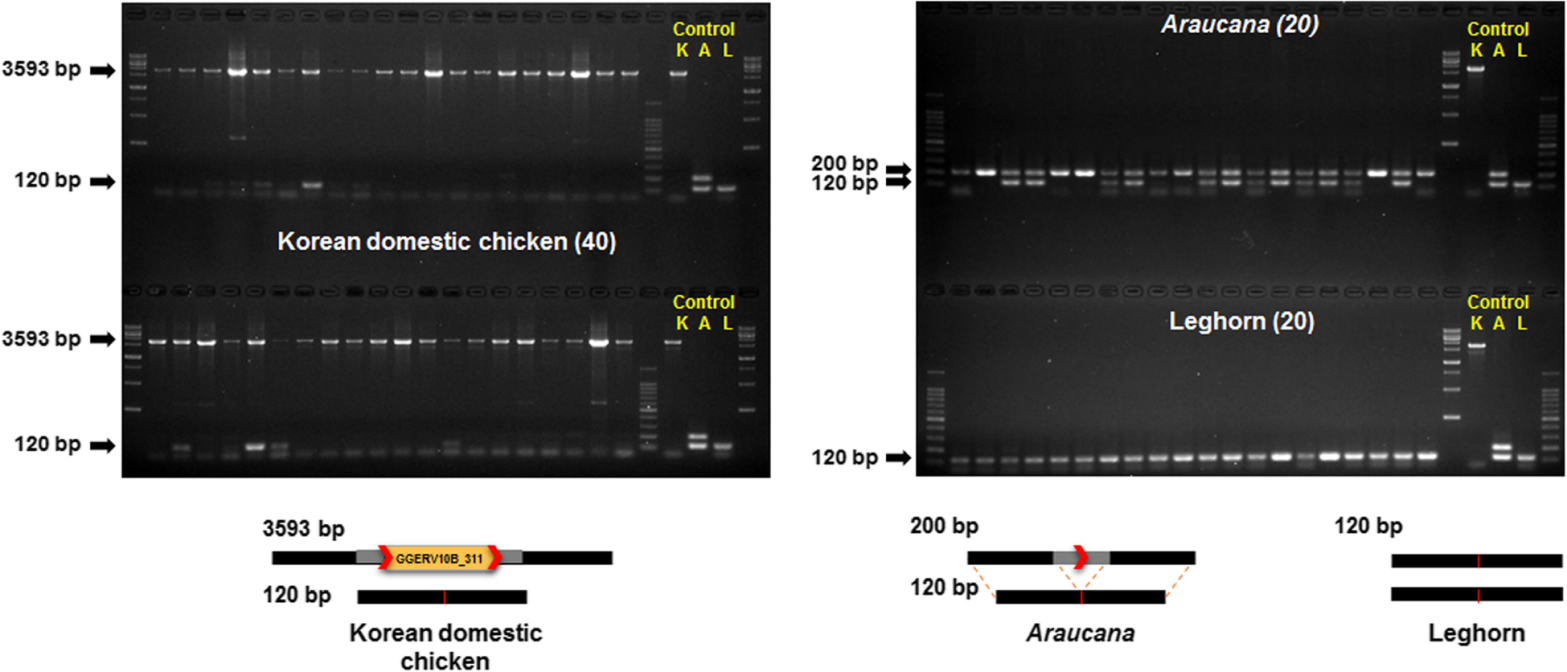
Polymorphic pattern of the GGERV10B_311 locus in three chicken breeds. PCR amplification was conducted with 80 chicken DNA samples from three different chicken breeds (40 Korean domestic chicken, 20 Leghorn, and 20 *Araucana*). GGERV10B_311 (3,593 bp) insertion was present only in Korean domestic chicken (*left*) and small deletion allele (120 bp) was also detected. Two amplicon of *Araucana* indicates the absence of the GGERV10B_311 element and small deletion. Additionally, leghorn has only small deletion (*right*). Korean domestic chicken (K), *Araucana* (A), and leghorn (L)

One of *Araucana*-specific GGERV10A insertions locates in the 5′ flanking region of *SLCO1B3* gene and is responsible for the blue eggshell color in *Araucana.* It suggests that GGERV10 elements could be served as a genetic marker [32]. It suggests the possibility that any of the full-length GGERV10 elements could be breed-specific locus. As our polymorphism test showed that three of the 49 full-length GGERV10 elements, GGERV10B_107, GGERV10B_193, and GGERV10B_311, are polymorphic in the chicken breeds, we further examined them using PCR with 80 chicken-DNA samples from three different chicken breeds (40 Korean domestic chicken, 20 Leghorn, and 20 *Araucana*). Through the PCR assay, we found that GGERV10B_107 and GGERV10B_193 elements are insertionally polymorphic in the 80 chicken-DNA samples (data not shown) while GGERV10B_311 locus had one more state, a deletion event at the pre-insertion site of the element. As shown in Fig. 2, GGERV10B_311 element is Korean domestic chicken breed-specific (Additional file 11: Table S7). In the *Araucana* samples, a polymorphic pattern was observed at the pre-insertion site of GGERV10B_311element; one of the two different PCR products was the expected size for the case where GGERV10B_311 element is absent but the other one was smaller than the expected size. The Leghorn breed produced only one type of the PCR products which were smaller than the expected size for the case without GGERV10B_311 insertion. To verify the unexpected PCR results at the GGERV10B_311 locus, we sequenced the PCR products and performed sequence alignment of the region (Additional file 12). The result found that 80 bp deletion event occurred in the pre-insertion site of GGERV10B_311 element and the GGERV10B_311 element is Korean domestic chicken-specific.

**Fig. 3:**
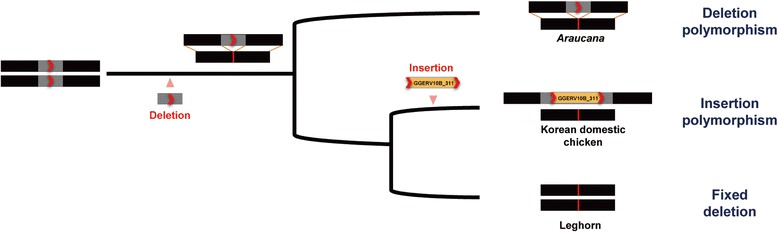
Schematic of incomplete lineage sorting in the GGERV10B_311 locus. The small deletion occurred before the divergence of the *Araucana* and other breeds and was still polymorphic at the time of speciation. Subsequently, the *Araucana* had maintained deletion polymorphism. After the divergence of the Korean domestic chicken and Leghorn, Korean domestic chicken-specific GGERV10B_311 element insertion event occurred and maintained insertional polymorphism. However, the deletion allele was fixed in the Leghorn species. The *Gray* box, *red* arrow, and *red* line indicate small deletion region, TSD, and deletion point, respectively

Incomplete lineage sorting events were previously reported to explain genetic polymorphism created by retrotransposons and retrotransposon-mediated deletions between closely related species [33–36]. In this study, a discordant PCR amplification pattern was shown at GGERV10B_311 locus, and incomplete lineage sorting between the three chicken breeds well explains the unexpected PCR result (Fig. 3). As shown in Additional file 13: Figure S3, a 80-bp deletion seemed to occur before the divergence of the Korean domestic chicken, Leghorn, and *Araucana* breeds. After the divergence of *Araucana* and the common ancestor of the Korean domestic chicken and Leghorn, the 80-bp deletion was still polymorphic in all of the three breeds. Then, the Korean domestic chicken was diverged from Leghorn, and the 80-bp small deletion was finally fixed in the Leghorn. Later, the GGERV10B insertion occurred only in the Korean domestic chicken breed. However, we cannot rule out that Leghorn species is artificially selected in farm due to modern commercial strain. Therefore, the evolution scenario could be modified or strongly supported if more chicken breeds are used in the further experiment.

## Conclusions

In this study, we characterized GGERV10 family, one of the youngest GGERV families in the chicken genome. The chicken reference genome contains a total of 593 GGERV10 elements but among them, only 49 elements are full-length. GGERV10 elements are retrotranspositionally inactive in the chicken genome because they are lack of intact genes necessary for the retrotransposition. However, they have a potential to regulate the expression of the neighboring genes as they retain 23 transcription factor binding sites. To identify breed-specific GGERV10 locus, the 49 full-length GGERV10 loci were subjected to a traditional PCR using 80 genomic DNAs isolated from the Korean domestic chicken, Leghorn, and *Araucana* as PCR template. Through the assay, GGERV10B insertion was identified to be Korean domestic breed-specific. This locus could be used to distinguish the Korean domestic chicken from other breeds of Leghorn and *Araucana*. This study supports that TEs including ERVs could be used as a molecular marker for species identification due to their virtually homoplasy-free phylogenetic character [37].

## Methods

### Computational analysis for GGERV10 loci of chicken (*Gallus gallus*)

To identify GGERV10 elements in the chicken genome, we extracted 593 GGERV10 loci from the Chicken reference genome (ICGSC Gallus_gallus-4.0/galGal4; Nov. 2011 assembly) by using UCSC Table Browser utility (http://genome.ucsc.edu/) and then, we identified full-length GGERV10 loci by RepeatMasker (http://www.repeatmasker.org/cgi-bin/WEBRepeatMasker). Finally, a total of 49 full-length GGERV10 loci were analyzed about their genomic features. First, we extracted each 10 kb sequences on 5′ and 3′ flanking region of full-length GGERV10 loci using the Chicken BLAT search Tool (https://genome.ucsc.edu/cgi-bin/hgBlat). Using these sequences, we calculated GC contents based on EMBOSS GeeCee server (http://emboss.bioinformatics.nl/cgi-bin/emboss/geecee). We also examined the gene density in the flanking sequences of the GGERV10 candidates. Each 2 Mb sequence of both flanking region of each GGERV10 locus was extracted and the number of genes were counted in these sequences using the National Center for Biotechnology Information Map Viewer utility (http://www.ncbi.nlm.nih.gov/mapview/map_search.cgi?taxid=9031&build=102.0).

### PCR amplification and sequence analysis

To confirm insertion of GGERV10 identified through computational analysis, we performed PCR in chicken genomic DNA panel. Chicken genomic DNA panel was composed of 9 chicken genomic DNA samples (3 Korean domestic chicken, 3 leghorn, and 3 blue-egg shell chicken). The panel was provided from National Institute of Animal Science (Korea). Oligonucleotide Primer set for PCR amplification of each identified GGERV10 locus was designed through Primer3 (http://bioinfo.ut.ee/primer3-0.4.0/primer3/) and Oligocalc (http://www.basic.northwestern.edu/biotools/oligocalc.html) programs. Primer information is summarized in Additional file 10: Table S6. PCR amplification was performed in 20 μL reaction volume using 10-20 ng template DNA, 200 nM of each oligonucleotide primer, and 10 μL of master mixture of 2X *EF Taq* Pre mix4 (SolGent, Seoul, Republic of Korea) containing DNA polymerase, PCR buffer, dNTP, tracking dye, and 5X Band Doctor™. PCR amplification was carried out by following process: an initial denaturation step of 5 min at 95 °C, followed by 35 cycles of 1 min at 95 °C, 40 sec at the optimal annealing temperature and optimal time depending on PCR product size for extension at 72 °C, followed by a final extension step of 10 min at 72 °C. Bio-rad™ iCycler thermocycler (Biorad, Munich, Germany) was used for PCR amplification. Amplified PCR products were loaded on a 1.5% agarose gel for electrophoresis, stained by EcoDye Nucleic acid staining solution (BIOFACT, Daejeon, Korea), and visualized with UV fluorescence. Four out of 49 GGERV10 candidates contains poly (N) stretches in the chicken sequence. So, these loci were sequenced and determined by using the BigDye Terminator v3.1 Sequencing Kit (Applied Biosystems, FosterCity, CA, USA) through ABI 3500 Genetic analyzer (Applied Biosystems).

### Phylogenetic analysis

To perform phylogenetic analysis, GGERV10 subfamily consensus sequences were generated using the module MegAlign available in the DNA Star program (DNA STAR Inc.,Wisconsin). And aligned GGERV10 elements with this consensus sequence using the software BioEdit version 7.0.5.3 (Hall, 1999). Molecular Evolutionary Genetics Analysis (MEGA) software 6 was used to construct phylogenetic tree using the neighbor-Joining method. Each node of the tree was estimated based on 1000 bootstrap. The bootstrap analysis was performed according to the Kimura-2-parpameter distance (Kimura, 1980).

Furthermore, to estimate evolutional age of each GGERV10 subfamily, full-length GGERV10 subfamilies were aligned based on LTR sequence except a few GGERV10 copies had partial truncated LTR. The putative age of each GGERV10 subfamilies were calculated with NETWORK 4.611 [23]. We used a nucleotide mutation rate of 0.2 ~ 0.26% per site per myr, assuming that ERVs accumulate mutations at the neutral evolution rate after their insertion.

### Transcription factor binding site search in GGERV10 LTR

To analyze putative transcription binding sites in consensus sequences of GGERV10 subfamily, we used TRANSFAC® Professional 7.4.1 (http://genexplain.com/transfac/) with threshold 0.95.

### RetroTector analysis

RetroTector10 program (http://retrotector.neuro.uu.se/pub/queue.php?show=submit), a platform-independent java program package, was used to investigate genomic structure of full-length GGERV10 candidates in the chicken genome. It includes three basic modules: (i) Prediction of LTR candidates, (ii) Prediction of chains of conserved retroviral motifs fulfilling distance constraints and (iii) Attempted reconstruction of the original retroviral protein sequences, combining alignment, codon statistics, and properties of protein ends.

## Additional files

Additional file 1: Table S1. Chromosome density of GGERV10 elements in the chicken genome. (XLSX 13 kb)
Additional file 2: Table S2. Summary of full-length GGERV10 elements. (XLSX 14 kb)
Additional file 3: Table S3. Summary of solo-LTR copies. (XLSX 41 kb)
Additional file 4: Figure S1. Alignment of LTR sequences between the full-length GGERV10 elements. Using the BioEdit program, 5′ and 3′ LTR sequences from 38 full-length GGERV10 elements were aligned. Shared sequences among five subfamilies were indicated by colored boxes (A, B, C, D, E, F, G, and H). Blue boxes of both ends indicate GGERV10 family-specific terminal inverted repeats of LTR region. Orange boxes in the ‘A’ region indicate 24-nt duplication. (TIF 11339 kb)
Additional file 5: LTR sequences of the full-length GGERV10 elements. (FAS 34 kb)
Additional file 6: Table S4. Age estimation of full-length GGERV10 elements. (XLSX 10 kb)
Additional file 7: Table S5. Investigation of full-length GGERV10 elements. (XLSX 18 kb)
Additional file 8: Figure S2. Investigation of putative transcription factor binding sites within the LTR sequence. Colored boxes indicate putative transcription factor binding sites in the LTR consensus sequence from GGERV10 subfamilies. Five GGERV10 subfamilies have shared or specific transcription factor binding sites. (TIF 10395 kb)
Additional file 9: LTR consensus sequences of each full-length GGERV10 subfamilies. (FAS 1 kb)
Additional file 10: Table S6. Primer information for the PCR amplification of full-length GGERV10 elements. (XLSX 16 kb)
Additional file 11: Table S7. Condition for the PCR amplification of GGERV10B_311 element. (XLSX 11 kb)
Additional file 12: Sequence alignment of GGERV10B_311 locus in three chicken breeds. (FAS 10 kb)
Additional file 13: Figure S3. Sequence comparison of GGERV10B_311 locus in three chicken breeds. Sequence alignment of GGERV10B_311 locus in three chicken breeds shows complex genomic feature. Purple boxes indicate primer sequences for GGERV10B_311 locus. Each colored box indicates three breeds: Korean domestic chicken (yellow), *Araucana* (green), and leghorn (blue). Blue box presents target site duplication (TSD) sequence. (TIF 8189 kb)

## Abbreviations

CR1: Chicken repeat1
*env*: Envelope gene
ERV: Endogenous retrovirus
*gag*: Group-specific antigen
GGERV: *Gallus gallus* endogenous retrovirus
LINE: Long Interspersed element
LTR: Long terminal repeat
MIR: Mammalian interspersed repeat
ORFs: Open reading frames
pbs: Primer-binding site
PCR: Polymerase chain reaction
*pol*: RNA-dependent DNA polymerase gene
*pro*: Protease gene
RT: Reverse transcriptase
TE: Transposable element
TSDs: Target site duplications
