# Supplementary figures and images for: Chicken (Gallus gallus) endogenous retrovirus generates genomic variations in the chicken genome
Source: Mob DNA. 2017 Jan 24;8:2. doi: 10.1186/s13100-016-0085-5 (PMC5260121; doi:10.1186/s13100-016-0085-5)

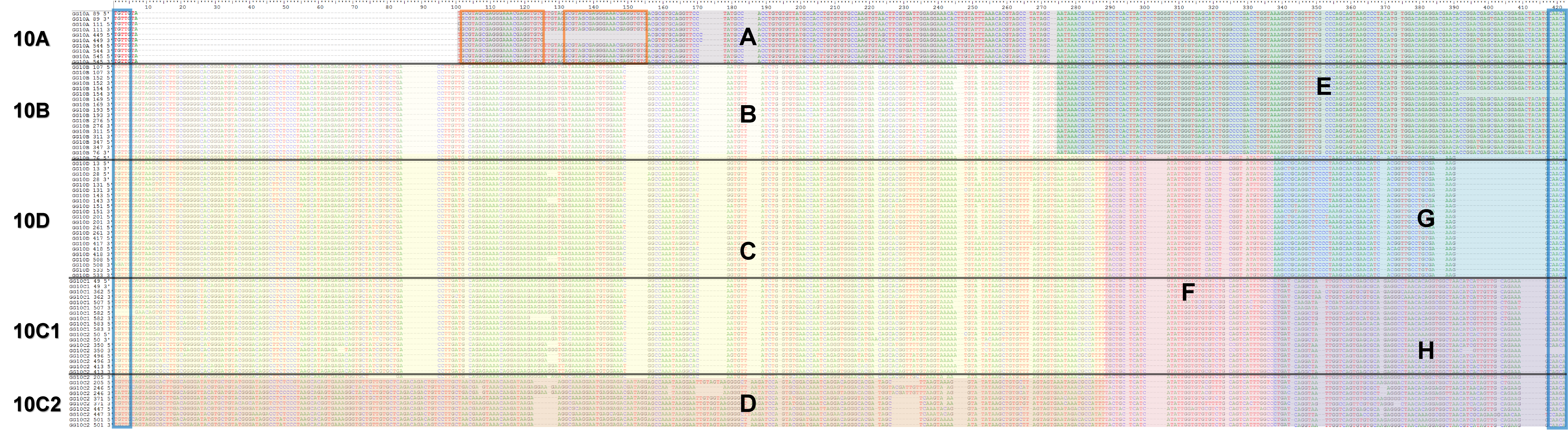

Supplement: Additional file 4: Figure S1. — Alignment of LTR sequences between the full-length GGERV10 elements. Using the BioEdit program, 5′ and 3′ LTR sequences from 38 full-length GGERV10 elements were aligned. Shared sequences among five subfamilies were indicated by colored boxes (A, B, C, D, E, F, G, and H). Blue boxes of both ends indicate GGERV10 family-specific terminal inverted repeats of LTR region. Orange boxes in the ‘A’ region indicate 24-nt duplication. (TIF 11339 kb) [file 13100_2016_85_MOESM4_ESM.tif]

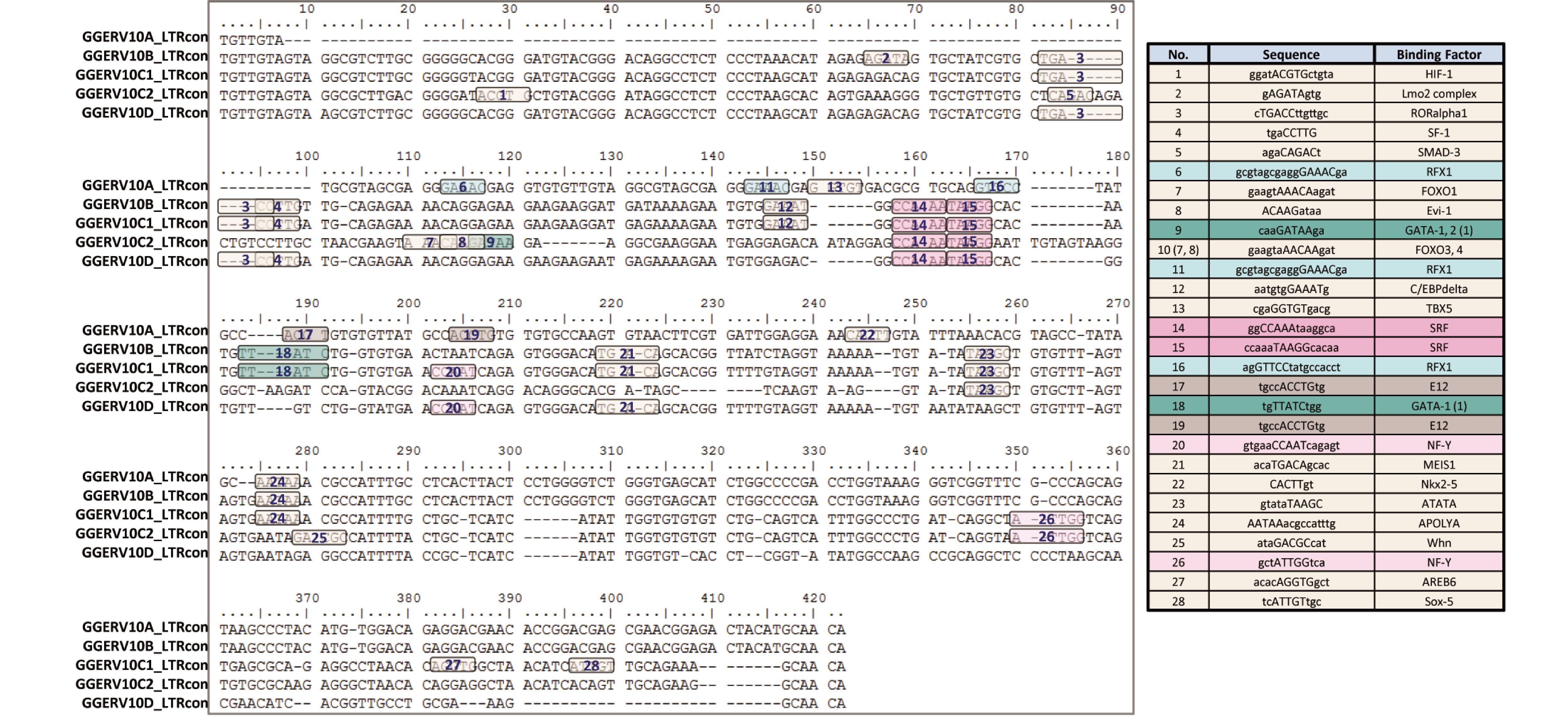

Supplement: Additional file 8: Figure S2. — Investigation of putative transcription factor binding sites within the LTR sequence. Colored boxes indicate putative transcription factor binding sites in the LTR consensus sequence from GGERV10 subfamilies. Five GGERV10 subfamilies have shared or specific transcription factor binding sites. (TIF 10395 kb) [file 13100_2016_85_MOESM8_ESM.tif]

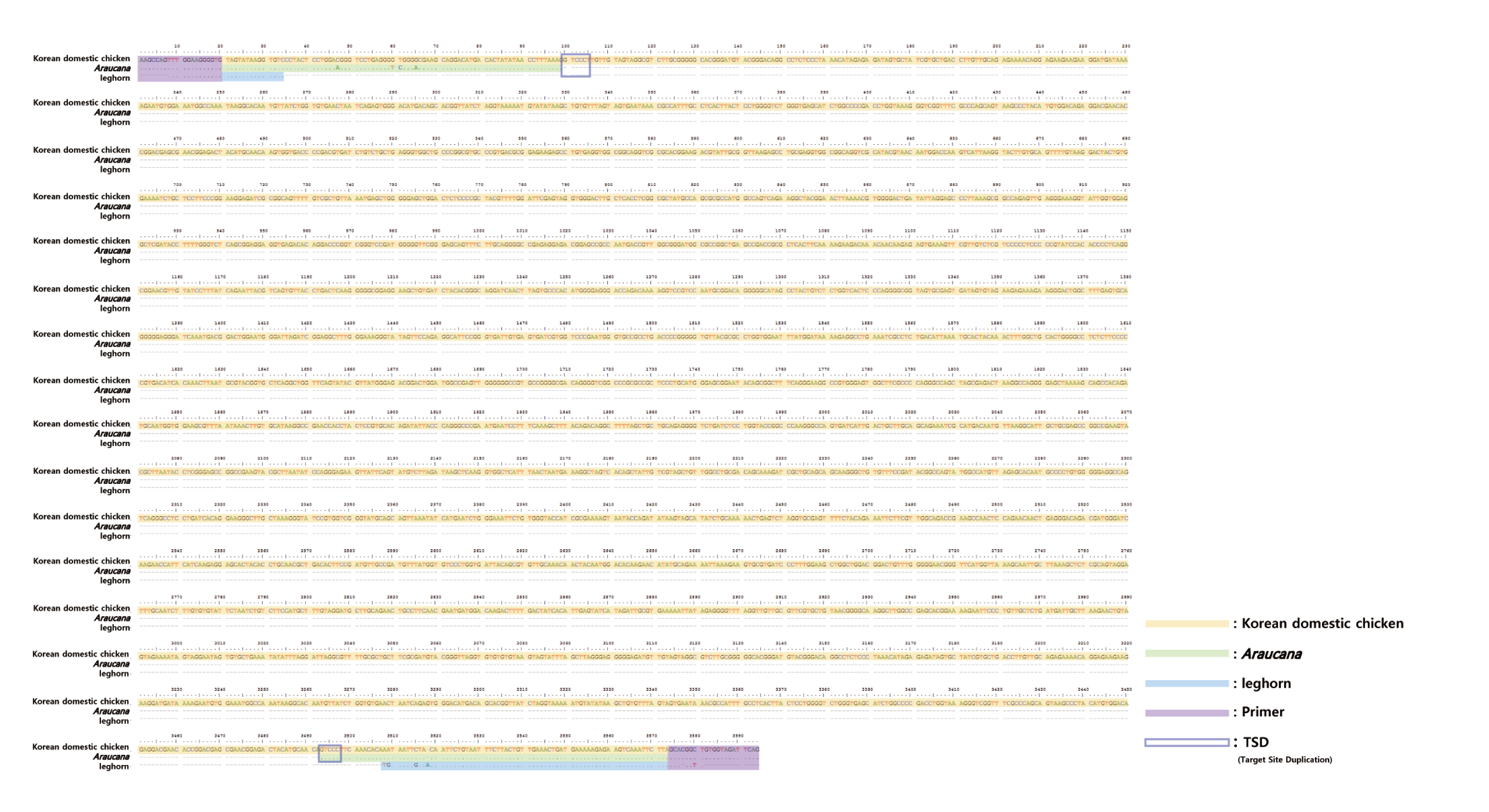

Supplement: Additional file 13: Figure S3. — Sequence comparison of GGERV10B_311 locus in three chicken breeds. Sequence alignment of GGERV10B_311 locus in three chicken breeds shows complex genomic feature. Purple boxes indicate primer sequences for GGERV10B_311 locus. Each colored box indicates three breeds: Korean domestic chicken (yellow), Araucana (green), and leghorn (blue). Blue box presents target site duplication (TSD) sequence. (TIF 8189 kb) [file 13100_2016_85_MOESM13_ESM.tif]
